# Rational Development of a Novel Hydrogel as a pH-Sensitive Controlled Release System for Nifedipine

**DOI:** 10.3390/polym10070806

**Published:** 2018-07-23

**Authors:** Fabián Avila-Salas, Yeray A. Rodriguez Nuñez, Adolfo Marican, Ricardo I. Castro, Jorge Villaseñor, Leonardo S. Santos, Sergio Wehinger, Esteban F. Durán-Lara

**Affiliations:** 1 Centro de Nanotecnología Aplicada, Facultad de Ciencias, Universidad Mayor, Huechuraba 8580000, Región Metropolitana, Chile; 2 BioNanoMaterials Lab|Drug Delivery and Controlled Release, Universidad de Talca, Talca 3460000, Maule, Chile; yrodriguez@utalca.cl (Y.A.R.N.); amarican@utalca.cl (A.M.); 3 Instituto de Química de Recursos Naturales, Universidad de Talca, Talca 3460000, Maule, Chile; jvillase@utalca.cl (J.V.); lssantos@utalca.cl (L.S.S.); 4 Multidisciplinary Agroindustry Research Laboratory, Universidad Autónoma de Chile, Talca 3460000, Maule, Chile; ricardo.castro@uautonoma.cl; 5 Carrera de Ingeniería en Construcción e Instituto de Ciencias Biomédicas, Universidad Autónoma de Chile, Talca 3460000, Maule, Chile; 6 Department of Clinical Biochemistry and Immunohematology, Faculty of Heatlh Sciences, Universidad de Talca, Talca 3460000, Maule, Chile; snunez@utalca.cl; 7 Center for Studies of Exercise, Metabolism and Cancer (CEMC), Universidad de Chile, Independencia 8380000, Región Metropolitana, Chile; 8 Departamento de Microbiología, Facultad de Ciencias de la Salud, Universidad de Talca, Talca 3460000, Maule, Chile

**Keywords:** drug release, crosslinking, nifedipine, cyclodextrin, swelling, thermogravimetric analysis, molecular simulation, interaction energy

## Abstract

This work depicts the rational development (in-silico design, synthesis, characterization and in-vitro evaluation) of polyvinyl alcohol hydrogels (PVAH) cross-linked with maleic acid (MA) and linked to γ-cyclodextrin molecules (γ-CDPVAHMA) as systems for the controlled and sustained release of nifedipine (NFD). Through computational studies, the structural blocks (PVA chain + dicarboxylic acid + γ-CD) of 20 different hydrogels were evaluated to test their interaction energies (Δ*E*) with NFD. According to the Δ*E* obtained, the hydrogel cross-linked with maleic acid was selected. To characterize the intermolecular interactions between NFD and γ-CDPVAHMA, molecular dynamics simulation studies were carried out. Experimentally, three hydrogel formulations with different proportions of γ-CD (2.43%, 3.61% and 4.76%) were synthesized and characterized. Both loading and release of NFD from the hydrogels were evaluated at acid and basic pH. The computational and experimental results show that γ-CDs linked to the hydrogels were able to form 1:1 inclusion complexes with NFD molecules. Finally, γ-CDPVAHMA-3 demonstrated to be the best pH-sensitive release platform for nifedipine. Its effectiveness could significantly reduce the adverse effects caused by the anticipated release of NFD in the stomach of patients.

## 1. Introduction

Nifedipine (NFD) is calcium channel blocker of the dihydropyridine type (see its chemical characteristics in Table 1) that is principally utilized for the treatment of hypertension, angina pectoris and in peripheral circulatory disorders such as Raynaud’s syndrome [1]. NFD is considered a suitable candidate for administration of release because of its characteristics: its short elimination half-life of no more than 4 h, its rapid absorption in the gastrointestinal tract and its ability to reduce blood pressure. Conventional formulation habitually is administered two or three times per day, which leads to large fluctuation in drug plasma concentration and adverse side effects in patients [2]. Due to the above, its relationship between short-acting calcium antagonists of dihydropyridines and the risk of myocardial infarction has become a topic of discussion among researchers. Short-acting calcium antagonists are involved in the increase in sympathetic nerve activation and reflex tachycardia. This increase may be one of the coronary risk factors in hypertensive patients (independent of pressure) [3].

Because of the undesirable side effects produced by standard treatments in patients, an innovative area based on drug delivery methods have arisen to reverse these adverse effects. The drug delivery systems offer numerous advantages if compared with conventional doses: improved efficacy, less toxicity and better quality of life of the patient [1]. Drug delivery systems include nanoparticles, dendrimers, liposomes and hydrogels, among others [5,6]. Among these drug release strategies, the hydrogels have received growing attention as biomaterials for drug delivery systems because of their biodegradable, biocompatible and tunable properties [7,8].

Porosity is the major feature of hydrogels, which can be tailored by adjusting the degree of network crosslinking in their scaffold, thus affecting their water absorption capacity [9,10,11]. The attraction of hydrogel towards water molecules determines a new key characteristic in the hydrogel called swelling degree [12,13]. This feature can aid in the loading of drugs into hydrogels during swelling and subsequent drug release during deswelling [14]. The drug release kinetics can be tuned through hydrogel properties such as polymer concentration, crosslink density, degradability, or drug–polymer affinity, among others [15].

On the other hand, several mechanisms have been elucidated to describe drug release from polymer hydrogel systems including diffusion, swelling, and chemically controlled release (erosion by enzymatic degradation and pH-responsive) [16]. This property offered by hydrogels for drug delivery applications involves the chance for sustained release, which results in keeping an appropriate local concentration of bioactive compounds over a long period [17]. Moreover, it is very important that the formulation based on hydrogels be harmless (non-allergic, non-toxic and biocompatible) [18].

Hydrogels have been generally limited to the delivery of hydrophilic drugs [19]. In this context, polyvinyl alcohol (PVA) [20,21,22], dicarboxylic acids (DCAs) [23] and cyclodextrins (CDs) [24,25] were selected as structural and functional components for designing the hydrogel. These three components have been approved by FDA [26,27] for use as biomaterials because their high biocompatibility. CDs were chosen since they can form inclusion complexes (host–guest type) with numerous types of insoluble molecules. The traditional types of CDs are formed of six, seven and eight D-glucopyranoside units (α-CD, β-CD or γ-CD, respectively) attached by R-1,4 bonds, allowing the possibility form hydrophobic cavities.

The overall proof of concept of this work was the rational development (in-silico design, synthesis, characterization and in-vitro evaluation) of three cross-linked polyvinyl alcohol hydrogels (PVAH) (cross-linked with maleic acid (MA)) and linked with different proportions of γ-CD molecules for each one. These formulations were named γ-CDPVAHMAs and they were evaluated as pH-sensitive release platforms for NFD. The efficiency of γ-CDPVAHMA to release NFD could be applied as a serious alternative in the non-conventional treatment of hypertension, angina pectoris and in peripheral circulatory disorders. Its effectiveness could significantly reduce the adverse effects caused by the anticipated release of NFD in the stomach of patients.

## 2. Materials and Methods

### 2.1. Computational Section

#### 2.1.1. Designing and Building of the Molecular Structures

The three-dimensional (3D) structures of NFD, DCAs, PVA monomer, γ-CD and acetic acid were designed and built through MarvinSketch software version 17.29.0, ChemAxon Ltd., Budapest, Hungary [28]. For all 3D structures, their protonation states at pH 3.0 and pH 7.4 were considered. The 3D geometries for each structure were optimized using Gaussian software version 16, revision A.03, Inc., Wallingford, CT, USA [29] at Density Functional Theory level using the B3LYP method and 6-311+G(d,p) as the selected basis set. The DCAs identified by Marican et al. 2018 [17] were used in this study.

#### 2.1.2. Inclusion Complexes (PVAchain-DA-γ-CD/NFD) Evaluation

A methodology that involves a Monte Carlo sampling [30] and semi-empirical quantum mechanical (SQM) [31] calculations was used to obtain the interaction energy (Δ*E*) for pair of molecules (Molecule 1–Molecule 2 complexes). The detail of the methodology was described in previous works [32,33,34]. Briefly, MOPAC2016 program version 16.111L for Linux, Colorado Springs, CO, USA [35] was used to obtain the Δ*E* through the following Equation (1):

Δ*E*_1,2_ = *E*_(Molecule 1–Molecule 2)_ − (*E*_(Molecule 1)_ + *E*_(Molecule 2)_) (1)

where “Molecule 1” represents the structural blocks (PVA chain + dicarboxilic acid + γ-CD) of 20 possible hydrogels and “Molecule 2” represents NFD. The difference between each block was the carboxylic acid. 20 different dicarboxilic acids were tested to see their influence in the calculation of Δ*E*. NFD molecule undergoes a protonation of its dihydropyridine ring nitrogen at acid pH (stomach pH); therefore, both structures (protonated and non-protonated) were considered for Δ*E* calculations. Due to the size of the Molecule 1–Molecule 2 complex and the associated computational cost to calculate the Δ*E*, only 20,000 pairs of conformations were considered for sampling.

#### 2.1.3. Building of Hydrogel-NFD Systems and Molecular Dynamic (MD) Simulation Study

Twenty-eight PVA chains (each 34 monomers long) were built with the LEAP program of AmberTools software version 17.05 for Linux, University of California, San Francisco, CA, USA [36]. These chains were distributed within a 3D orthorhombic box of 80 Å × 70 Å × 80 Å (*X*, *Y* and *Z* axes) using PACKMOL software version 16 for Linux [37] (considering a distance of 6 Å separating the chains from each other). To model the 10:2 proportion of PVA:MA, the polymer matrix built with the 28 PVA chains (considering a total of 952 PVA monomers) was crosslinked incorporating 190 MA into the polymer matrix, of which 150 were covalently bonded to the –OH groups of PVA chains. The crosslinking procedure was performed according to previous work described by Marican et al. (2018) and Avila-Salas et al. (2018) [17,34]. Subsequently, LEAP program was used to covalently bonding 16 γ-CD molecules to the MAs that were linked to the PVA only by one end. PACKMOL program version 16 for Linux [37] was used to incorporate 30 NFD molecules around each crosslinked hydrogel (randomly and considering a separation distance of 8 Å, both between them and with the polymer). The full polymer systems (hydrogel + NFD) were added in the center of a 140 Å × 130 Å × 140 Å (axes *X*, *Y*, *Z*, respectively) box, which was solvated with methanol using the “System Builder” Module of Desmond/Maestro software version 2017-4 academic release for Linux, DE Shaw Research, New York, NY, USA [38].

Three MD simulations were carried out for about 50 ns each, all them considered the default relaxation protocol used by Desmond/Maestro software version 2017-4 academic release for Linux, DE Shaw Research, NY city, USA [38] to prepare the system before the simulation. Major details were described in previous work by Marican et al. (2018) and Avila-Salas et al. (2018) [17,34]. The first MD simulation was carried out for 50 ns considering the system inserted in a box of methanol. From the final trajectory, the polymer matrix and the NFD molecules bound to it (or located at a distance less than 5.5 Å) were extracted.

The second simulation used the extracted frame. To simulate the behavior of the polymer system with NFD in an environment of acid pH (stomach pH), two extra steps were carried out: first the NFD nitrogens of the dihydropyridine rings were protonated, and, second, all the carboxyl groups of MA not linked to PVA were also protonated (-COOH groups). To simulate an acetate buffer (pH 3.0), PACKMOL software version 16 for Linux [37] was used to incorporate acetic acid molecules to the solvent box. Then, both sodium ions and TIP3 water molecules were incorporated the solvent box with the “System Builder” Module of Desmond/Maestro software version 2017-4 academic release for Linux, DE Shaw Research, New York city, NY, USA [38]. Finally, the MD simulation was carried out for 50 ns. As in the first simulation, from the final trajectory, the polymer matrix and the NFD molecules bound to it (or located at a distance less than 5.5 Å) were extracted.

For the third simulation, the above extracted frame was used. To simulate the behavior of the polymer system with NFD in an environment of neutral-basic pH (intestinal pH), a series of steps were carried out: first the NFD nitrogens of the dihydropyridine rings were deprotonated, and then, all the carboxyl groups of MA not linked to PVA were also deprotonated (-COO^−^ groups). To simulate a PBS buffer (pH 7.4), PACKMOL software version 16.070.3 for Linux [37] was used to incorporate phosphate molecules to the solvent box. Then, both sodium ions and TIP3 water molecules were incorporated the solvent box with the “System Builder” Module of Desmond/Maestro software version 2017-4 academic release for Linux, DE Shaw Research, New York, NY, USA [38]. Finally, the MD simulation was carried out for 50 ns. The parameter details for the MD simulations were described in previous work by Marican et al. (2018) and Avila-Salas et al. (2018) [17,34].

One thousand frames were extracted from MD simulations to carry out the intermolecular interactions analysis and the characterization of their structures. The Radius of Gyration (RGYR) [39] and the Solvent Accessible Surface Area (SASA) [40] were calculated (Scripts S1 and S2, respectively). TCL script (Script S3) of VMD software version 1.9.2 for Linux [41] was used to quantify the number of NFD molecules located at a distance less than 5.5 Å from the hydrogel. All results of the trajectories analysis of SASA, RGYR and the NFD capture calculations were analyzed and plotted using Gnuplot program version 5.2 [42]. BIOVIA Discovery Studio Visualizer version 2017 R2 (for Windows), Accelrys Software Inc., San Diego, CA, USA [43] was used to analyze the intermolecular interactions between hydrogel and NFD molecules.

### 2.2. Experimental Section

#### 2.2.1. Materials

Polyvinyl alcohol (PVA) 30–60 KDa (a water-soluble synthetic polymer), maleic acid (MA) (dicarboxylic acid), γ-cyclodextrin polymers (γ-CD), nifedipine (NFD) analytical standards, (3,4,5-dimethylthiazol2-yl)-2-5-diphenyltetrazolium bromide (MTT), dimethyl sulfoxide (DMSO), acetonitrile (HPLC grade), NaHCO_3_ and reagents to prepare phosphate buffer saline (PBS) (pH 7.4), and acetate buffer (pH 3.0) were purchased from Sigma-Aldrich (St. Louis, MO, USA). HCl (HPLC grade) was purchased from Merck (Darmstadt, Germany).

#### 2.2.2. Synthesis of γ-CDPVAHMAs

**Figure 1:**
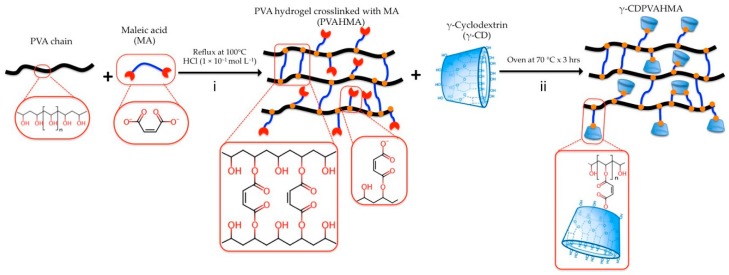
Schematic depiction of γ-CDPVAHMA (Polyvinyl alcohol hydrogel cross-linked with maleic acid and also linked with γ-cyclodextrin molecules). General syntheses were divided into two phases. First phase (pre-gel solution): Crosslinking reaction of PVA by esterification using MA as crosslinking agent. Second phase: Crosslinking reaction of PVA-MA (pre-gel solution) with γ-CD. Scheme was based on the process published by Marican et al. (2018) [17].

γ-CDPVAHMAs with three different proportions (*w*/*w*) of γ-CD were produced. The development of these formulations was performed through the esterification of PVA and MA according to Marican et al. (2018) [17] (Figure 1). Succinctly, the syntheses were carried out in distilled water using PVA with MA (20 wt%) as starting material compounds and 1 × 10^−1^ mol L^−1^ HCl (pH 1) as the catalyst. The reaction was agitated and kept under reflux in a necked flask at 100 °C (oil bath) for 3 h. The chemical reaction mixture was separated into three equal portions and placed in three glass vials, and a different amount of γ-CD was added to each one. The final γ-CD concentration for each formulation was 2.43, 3.61 and 4.76 wt%, termed as γ-CDPVAHMA1, γ-CDPVAHMA2, and γ-CDPVAHMA3, respectively. Next, every reaction was placed in an oven at 70 °C for 4 h to complete the crosslinking. Subsequently, γ-CDPVAHMA1, γ-CDPVAHMA2, and γ-CDPVAHMA3 were washed three or four times with a saturated sodium bicarbonate solution and once with distillated water to remove the excess acid. Lastly, each formulation obtained was lyophilized to afford a xerogel.

#### 2.2.3. Swelling Evaluation

The swelling studies or water uptake process was evaluated through equilibrium swelling ratio (%ESR) at desired time intervals as stated in the following description: 50 mg of dried hydrogel membrane discs of 0.5 mm thickness and 1 cm diameter were immersed in PBS (pH 7.4) and acetate buffer (pH 3.0) at 25 °C to swell. At regular time intervals (between 0 and 22 h), the swollen hydrogel was taken out from the swelling medium to weigh after removal of the surface water with filter paper. Finally, the hydrogel was put in the same vial with the respective solution. The readings were continually registered until a constant weight was reached. The swelling degree was calculated as %ESR applying Equation (2):

(2) $$\%\mathrm{ESR}=\frac{M_{h}-M_{x}}{M_{h}}\times 100$$

where %ESR is swelling index or equilibrium swelling ratio, *M_h_* is the mass of the swollen hydrogel, and *M_x_* is the mass of the xerogel.

#### 2.2.4. Fourier-Transform Infrared (FT-IR) Study

FT-IR spectra of γ-CDPVAHMAs were recorded on a Nicolet Nexus 470 spectrometer (Thermo Scientific, Waltham, MA, USA) inside the region of 4000–400 cm^−1^ accumulating 32 scans per spectrum at an optimum resolution of 4 cm^−1^. The spectra were acquired in KBr pellets.

#### 2.2.5. Thermal Gravimetric Analysis (TGA)

The analysis of thermic stability of all hydrogels was performed in a thermogravimetric analyzer TGA-Q500 (TA Instruments, New Castle, DA, USA). The dried samples of 5–10 mg were analyzed and then placed into a Pt crucible. After, they were heated at a constant rate of 20 °C min^−1^ from room temperature to 600 °C in air as a reactive gas with a mass flow of 60 mL min^−1^. In addition, 40 mL min^−1^ of N_2_ was used as protection gas in the electronic balance. All the analysis deconvoluted Gaussian peaks in the thermogram (DTG) were calculated using OriginPro software (OriginLab, OriginPro 8.5, USA).

#### 2.2.6. NFD Loading in γ-CDPVAHMA1, γ-CDPVAHMA2, and γ-CDPVAHMA3

The loading efficiency of NFD on charged γ-CDPVAHMA1, γ-CDPVAHMA2, and γ-CDPVAHMA3 was assessed obtaining the difference in mass utilizing Equation (3):

NFD content (mg) = X_0_ − X_NDP_ (3)

where X_0_ and X_NFD_ are the mass of xerogel without NFD and with NFD, respectively. To load the drug in the matrix of formulations, an aqueous solution of 0.08 mg mL^−1^ of NFD in PBS at pH 7.4 was utilized. Thus, the γ-CDPVAHMAs (50 mg each) were used per experiment, utilizing 10 mL of aqueous solution for every experiment. The γ-CDPVAHMAs were placed in a shaker (120 rpm) at room temperature for 24 h. The drug was stored in an amber glass container wrapped with aluminum foil and kept in a refrigerator at 5–7 °C. During the entire analysis, the NFD was manipulated in an amber glass container wrapped with aluminum foil as well.

#### 2.2.7. Drug Release Evaluation of γ-CDPVAHMA1, γ-CDPVAHMA2 and γ-CDPVAHMA3

The prepared γ-CDPVAHMA formulations are presented in Table 2. Concisely, the systems were loaded with a NFD aqueous solution in PBS (0.08 mg mL^−1^). The formulation without the drug served as the negative control. Thus, pre-weighed dried hydrogels (from each formulation) were loaded by immersion into the vial with 5 mL of PBS (pH 7.4). The vials with the samples were incubated in a shaker incubator water bath (Farazteb, Iran) at 37 °C ± 0.2 °C and shaken at 40 ± 2 rpm. At specific time intervals, a 1.0 mL sample solution was obtained from the release medium, which was substituted with the same volume of pure PBS. The NFD samples were evaluated by a Perkin Elmer series 200 HPLC apparatus (Norwalk, CT, USA). A C-18 Kromasil 100-5-C18 (250 mm × 4.6 mm i.d. × 5 mm) column and a UV-Vis detector was utilized for the analysis of eluents. The chromatographic conditions were the following: mobile phase, as isocratic elution (20 mM PBS/Acetonitrile (42:58, *v*/*v*)) at a flow rate of 1.0 mL min^−1^ and at ambient temperature. The injection volumes used were 50 uL from each filtered sample, and the wavelength selected for evaluation was at 240 nm.

The NFD cumulative release from each formulation was obtained by applying the relation between the amounts of released and absorbed NFD through Equation (4):

(4) $$\%\;\mathrm{NFD}\;\mathrm{cumulative}\;\mathrm{release}=\frac{\mathrm{Amount}\;\mathrm{of}\;\mathrm{released}\;\mathrm{NFD}}{\mathrm{Amount}\;\mathrm{of}\;\mathrm{absorbed}\;\mathrm{NFD}}\times 100$$

#### 2.2.8. Cell Viability Assay

Cytotoxicity and viability of fibroblasts cells was measured utilizing MTT assay [44]. Specifically, the cells were placed in 24-well plates (1.6 × 10^4^ cells per well, approximately). Afterward, the cells (5 μL) and Dulbecco’s Modified Eagle Medium (DMEM)-High medium (150 μL) were added and incubated at 37 °C in 5% CO_2_ for 24 h. Next, the medium was substituted by fresh DMEM-High (100 μL) per well containing concentrations of γ-CDPVAHMA1, γ-CDPVAHMA2, and γ-CDPVAHMA3 of exactly 500, 1000, 1500, 2000 and 2500 μg mL^−1^ per hydrogel. Fresh medium without formulation served as the negative control. After 24 h, the cell viability was calculated. Briefly, a volume of MTT solution (5 μL, 3 mg mL^−1^ in PBS) and a volume of pure medium (50 µL) were added to each sample and incubated in the dark at 37 °C for 4 h; formazan crystals were then solubilized in DMSO (100 µL) for 18 h. Then, the optical density (OD) of each solution (supernatant) was read at 570 nm (Spectrophotometer, Packard Bell, Meriden, CT, USA). Cytotoxicity of each formulation was expressed as percentage of viability with regard to untreated control cells (the mean o.d. of untreated cells was established as 100% viability).

#### 2.2.9. Statistical Analysis

An experimental design, based on 2N, was used to evaluate the optimal experimental conditions for the release of NFD by the formulations of synthesized hydrogels. The variables under study were Time, pH, and proportion of CD. These variables were coded between −1 and 1, to give them the same statistical weight. The results were expressed as averages ± standard deviations (*n* = 3).

## 3. Results and Discussions

### 3.1. Inclusion Complexes (γ-CD/NFD) Study Through Interaction Energies Calculations

Semi-empirical quantum mechanical (SQM) calculations were used to obtain the interaction energy (Δ*E*) for Molecule 1–Molecule 2 complexes. “Molecule 1” represents the structural blocks (PVA chain + dicarboxilic acid + γ-CD) of 20 possible hydrogels and “Molecule 2” represents NFD. NFD molecules undergoes a protonation of its dihydropyridine ring nitrogen at acid pH (stomach pH), therefore, both structures (protonated and non-protonated) were considered for Δ*E* calculations. Table 3 shows the values of the average interaction energies calculated. The results indicate that the block with Maleic Acid (MA) showed the greatest difference of interaction energy (−1.97 kcal mol^−1^) when it passed from an acidic environment to a basic environment. Therefore, it would be a good candidate for the process of crosslinking the PVA hydrogel, because it would allow the affinity of the hydrogel to be varied by NFD in a specific pH environment. The blocks that showed a lower variance of interaction energy between environments would not allow manipulation of NFD release at a specific pH.

**Figure 2:**
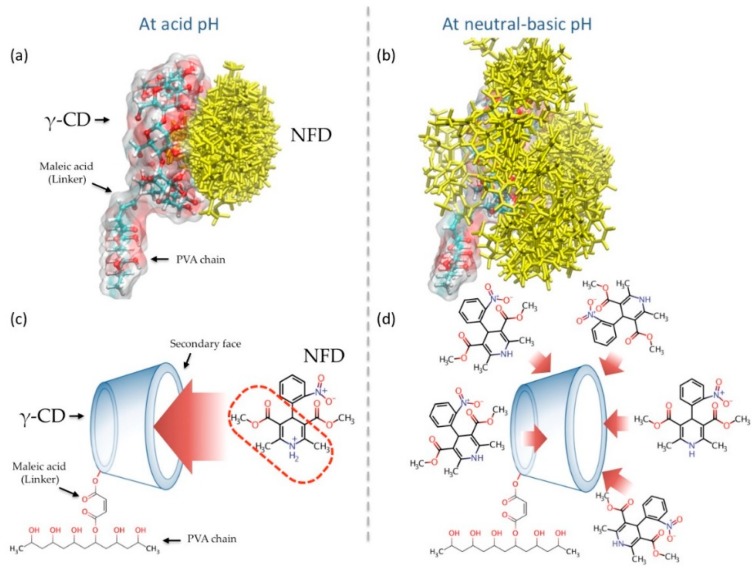
(**a**,**b**) Spatial distribution of the 200 conformations with the best interaction energy for the complexes: PVAchain-MaleicAcid-γ-CD/NFD at acid and neutral-basic pH, respectively. (**c**,**d**) Representative description of the inclusion processes that led to the formation of the γ-CD/NFD complex at acid and neutral-basic pH, respectively.

When the geometries of the 200 γ-CD/NFD conformations with the best interaction energies were analyzed, it was observed that at acid pH the NFD molecules entered the interior of the γ-CD cavity in all cases. The protonated structure of NFD generated a stable inclusion complex (Figure 2a,c). When the NFD molecule undergoes deprotonation when subjected to a neutral or basic pH, the affinity γ-CD/NFD was lost and it was not possible to form an inclusion complex (Figure 2b,d).

### 3.2. Molecular Dynamics (MD) Simulations Studies

MD simulations were carried out to characterize the structures of γ-CDPVAHMA hydrogels and their intermolecular interactions with NFD molecules at acid and neutral-basic pH. To achieve this, the systems (hydrogels and NFD molecules) were immersed in different solvent boxes to mimic the environment at both pH. The MD simulations were performed for about 50 ns of simulation each.

**Figure 3:**
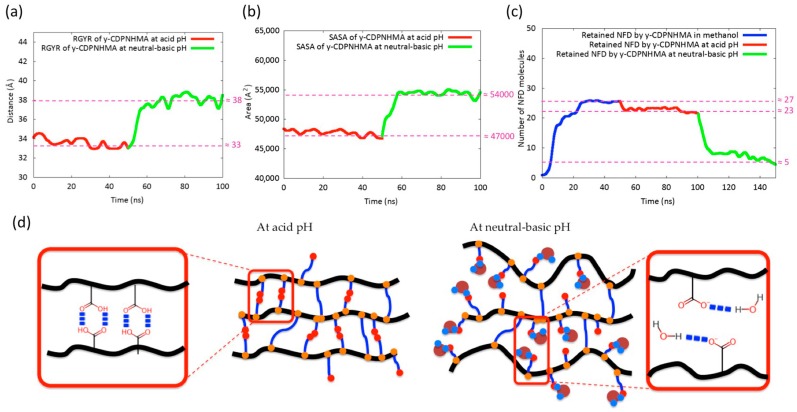
(**a**,**b**) Radius of Gyration (RGYR) and Solvent Accessible Surface Area (SASA) plots of the γ-CDPVAHMA at acid (0–50 ns) and neutral-basic pH (51–100 ns), respectively. (**c**) Number of NFD molecules retained by γ-CDPVAHMA during the three simulations: in methanol (0–50 ns), at acid pH (51–100 ns) and at neutral-basic pH (101–150 ns). (**d**) Schematic representing the possible interactions between carboxylic groups at acid pH (formation of hydrogen bonds) and hydrogen bonds between deprotonated carboxylic groups and water molecules at neutral-basic pH.

Figure 3a shows the behavior of the RGYR of both hydrogels in the MD simulations. γ-CDPVAHMA at acid pH showed a lower RGYR than at neutral-basic pH. This could be because the carboxyl groups of MA not linked to PVA are protonated (–COOH groups) and they are able to generate hydrogen bonds with the others MA connected to the PVA chains, resulting in stabilization and structural compacting of the hydrogel due to the attraction forces between PVA chains produced by these bonds (Figure 3d). When the hydrogel is subjected to a more basic environment, the carboxyl groups are deprotonated, the PVA chains are separated (repulsive forces between PVA chains) and their affinity for the water molecules increases (Figure 3d), the polymer network absorbs water through hydrogen bonds formed between water molecules and the deprotonated carboxyl groups. This is in accordance with the SASA graph, which shows how the hydrogel subjected to basic pH increases its solvent accessible area (Figure 3b).

**Figure 4:**
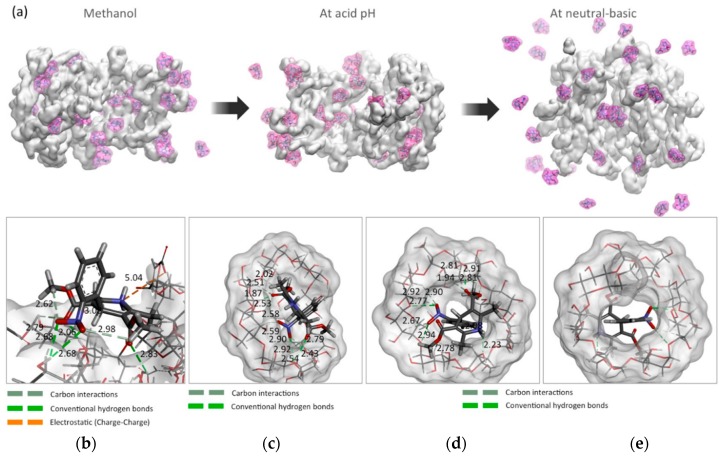
(**a**) Snapshots of the final results of each molecular dynamics simulation between γ-CDPVAHMA and NFD in methanol and in environment of acid and neutral-basic pH. Snapshots of the main intermolecular interactions generated between γ-CDPVAHMA and NFD: (**b**) in superficial nano-cavities of the hydrogel; (**c**) with the hydrophobic cavities of γ-CD added to the hydrogel; and (**d**,**e**) front and back face of the γ-CD/NFD inclusion complex.

The ability of the hydrogel to capture and retain NFD molecules during the first 100 ns of simulation is shown in Figure 3c. It was considered a contact distance of 5.5 Å between the polymer chains and NFD molecules. At acid pH (50–100 ns of simulation), the hydrogel retains more than 90% of the NFD molecules (Figure 4a). The main interactions that keep them together are hydrogen bonds and electrostatic interactions (charge-charge), mainly with γ-CD molecules, with which it is able to form highly stable inclusion complexes in this environment (Figure 4c–e). Notwithstanding the above, the degree of compaction of the hydrogel allows the generation of surface nano-cavities capable of attaching NFD molecules (Figure 4b).

When the hydrogel was subjected to a more basic environment (101–150 ns of simulation), the compact structure of the hydrogel changed, its interior was solvated with more water molecules (its hydrophilicity increases) and the γ-CD/NFD inclusion complexes were destabilized. The latter could also be caused by deprotonation in the nitrogen located in dihydropyridine ring of NFD.

The efficiency of γ-CDPVAHMA to controlled release NFD to a specific pH environment could be applied as a serious alternative to the conventional form of intake of this drug.

To compare how the choice of specific linker affects the swelling and release capacity of NFD of different hydrogels, SASA and NFD retention studies have been carried out for hydrogels crosslinked with malic acid (MLA) and glutamic acid (GLU). MLA is the second best candidate according to the energy of interaction calculated in the blocks (Table 3) and GLU is the worst of the whole series analyzed, because it presents a minimum variation of interaction energy when it goes from an acidic environment to a basic one. Figure S1 shows that the hydrogel crosslinked with malic acid (γ-CDPVAHMLA) has a similar behavior (SASA and NFD retention) to the hydrogel crosslinked with maleic acid (γ-CDPVAHMA) at acid pH; however, at basic pH, there is a difference which generates a lower release of the drug to the environment. In the case of the hydrogel crosslinked with glutamic acid (γ-CDPVAHGLU) the difference is minimal when it passing from one pH to another, there is practically no conformational change, the SASA remains constant and the efficiency in the NFD release at basic pH drops up to 40%.

### 3.3. Preparation of γ-CDPVAHMAs

The preparations of γ-CDPVAHMAs were made as exemplified in Figure 1. Concisely, the hydrogel films were synthetized using polymerization by esterification in the presence of HCl as a catalyst. The esterification process is simply a condensation of the hydroxyl group from PVA and carboxylic acid of MA. Once the pre-hydrogel was produced, γ-CD was incorporated where the hydroxyl groups from γ-CD were esterified with carboxyl groups still available from MA. The characterization analysis from FT-IR and TGA established the conjugation between γ-CD and MA into the hydrogel. As reported by Schanuel et al. (2015) [6], a 10:2 crosslinking of PVA:Maleic acid was selected because it generates a good porosity in the hydrogel [6]. Additionally, to improve its activity, the content of γ-CD was varied according to what is shown in Table 2.

### 3.4. ESR Results

**Figure 5:**
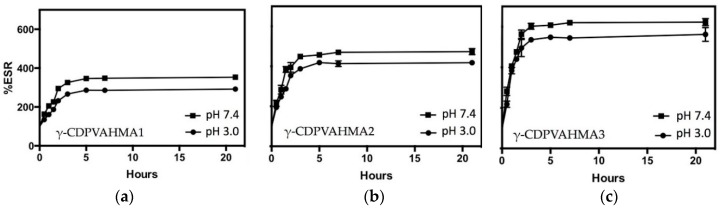
Dependence of the %ESR of γ-CDPVAHMA hydrogels on the amount of γ-CD and pH: (**a**) %ESR of γ-CDPVAHMA1 at pH 3.0 and pH 7.4; (**b**) %ESR of γ-CDPVAHMA1 at pH 3.0 and pH 7.4; and (**c**) %ESR of γ-CDPVAHMA1 at pH 3.0 and pH 7.4.

As previously mentioned, the swelling degree is a key factor in the loading and subsequent drug release from the hydrogel platform. Consequently, as proof of concept, it is highly important to evaluate the swelling degree behavior of the hydrogel under physiological pH (7.4) and stomach pH (2.0–3.0). Therefore, experiments were performed with the aim of evaluating the swelling capacity of the synthesized hydrogels with different γ-CD percentages at pH 7.4 and 3.0 at room temperature. Figure 5 shows the swelling degree for the three different formulations depicted in Table 2. This figure displays that the swelling degree increased considerably with time for all γ-CDPVAHMAs (in both pH models). For the three γ-CDPVAHMAs, the swelling degree in the first phase increased quickly and then slowly (after 5 h). This conduct was because the hydrogels achieved a constant and maximum swelling. In both pH models, γ-CDPVAHMA1, γ-CDPVAHMA2 and γ-CDPVAHMA3 reached the swelling equilibrium (zero order) at about 4–5 h. A significant difference for the all cases was observed between the two pH models. For instance, γ-CDPVAHMA1 exhibited a better swelling degree at pH 7.4 with a value of approximately 330%, while at pH 3.0 the swelling degree was about 250%.

**Figure 6:**
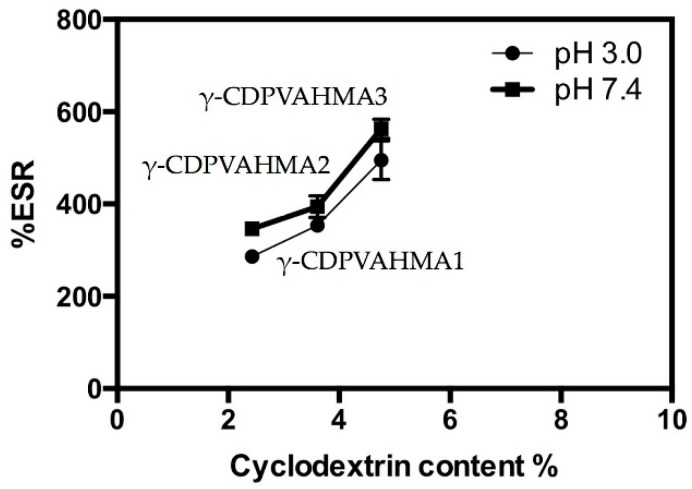
%ESR of γ-CDPVAHMA1, γ-CDPVAHMA2, and γ-CDPVAHMA3 at pH 3.0 and 7.4 with respect to time.

The same behavior was observed with γ-CDPVAHMA2 and γ-CDPVAHMA3, where the swelling degree at pH 7.4 and 3.0 were around 450% and 400%, and 600% and 500%, respectively, as illustrated in Figure 5. The ESR for the three different formulations is shown in Figure 6. This figure shows that the swelling index increased significantly with time for the set of γ-CDPVAHMAs and at both pH models. As has been reported in previous works, the swelling degree has a direct relationship with the structural nature of the polymer and its properties, including the average molecular weight, the rigidity of the polymer chain, the degree of crosslinking, network mesh size and external conditions, such as temperature and pH [13,45]. Furthermore, the swelling degree observed could depend on the absorption mechanism, which is determined by the diffusion process of water into the pores of hydrogel [46].

The synthesized hydrogels in this work present pH-dependent swelling behavior due to ionic networks. These ionic networks contain acidic pendant groups contributed by the MA with two kinds of pKa (pKa1 = 1.94 and pKa2 = 6.22) [47]. In aqueous media of appropriate pH and ionic strength, the pendant groups are ionized developing fixed charges on the hydrogel. Typically, changing conditions such as temperature and pH, among others, results in a homogeneous expansion or contraction of hydrogels in all directions [48]. The swelling degree in all cases is higher at pH 7.4 than 3.0 in these hydrogels; therefore, the ionization occurs when the environment pH is above the pKa of the ionizable group (at pH 7.4). As the ionization degree increases (increased pH of the system from 3.0 to 7.4), the number of fixed charges increases, causing an increased electrostatic repulsion between chains into the networks. Due to the above, network hydrophilicity increases and boosts swelling degree [49]. Therefore, in this case, when increasing the pH, the electrostatic repulsions produced that the uptake of solvent into the network increased and the hydrogel expanded. On the other hand, the incorporation of γ-CD into the hydrogels leading to the increase in ESR as shown in Figure 5, which could be explained by the several hydroxyl groups on γ-CD forming hydrogen bonds with water molecules [17].

### 3.5. NFD Loading and In Vitro Release Behavior of γ-CDPVAHMAs

Table 4 presents the amount of NFD loaded into the γ-CDPVAHMA hydrogels. There was no statistically significant difference between the results obtained.

### 3.6. Photograph Analysis: Sample Preparation and Viewing

**Figure 7:**
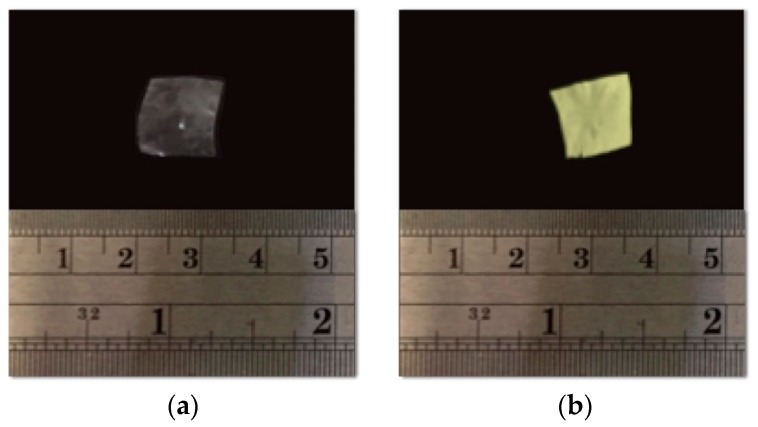
Photographs: (**a**) a small fragment of xerogel γ-CDPVAHMA3 without NFD; and (**b**) of small fragment of xerogel γ-CDPVAHMA3 with NFD. The formulations were lyophilized after the swelling process.

Photographs were used to compare the macromorphology of the dried samples; γ-CDPBHMA3 without NFD and γ-CDPVAHMA3 with NFD are depicted in Figure 7. Figure 7a,b shows photographs of γ-CDPVAHMA3 without and with NFD, respectively. The presentation of γ-CDPVAHMA3 without NFD was transparent unlike the yellowish appearance of γ-CDPVAHMA3 with trapped NFD.

The γ-CD plays two key roles in these formulations. The first is to form a complex with NFD when the hydrogel is loaded with the drug. Second, since NFD is an extremely photosensitive drug, it requires restricted protection from light during manufacturing, storage, and handling during its consumption. Inclusion complexation of NFD with γ-CDs could be beneficial in protecting the drug against the effect of light [50].

**Figure 8:**
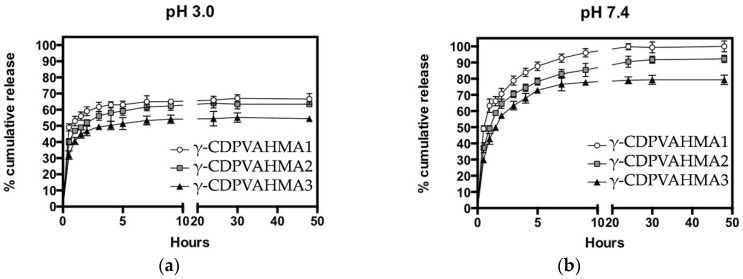
NFD release profile from γ-CDPVAHMA formulations in two model solutions, pH 7.4 and pH 3.0: (**a**) cumulative release from γ-CDPVAHMA1, −2, and −3 at pH 3.0; and (**b**) cumulative release from γ-CDPVAHMA1, −2, and −3 at pH 7.4.

In vitro NFD cumulative release from the NFD-loaded γ-CDPVAHMAs was investigated by monitoring the amounts of cumulative released NFD from three different NFD-loaded γ-CDPVAHMA formulations in two-model solution mixtures at pH 7.4 and pH 3.0 at 25 °C as a function of time. The results reported here are interesting according to the kinetic release of NFD. As shown in Figure 8a, at pH 3.0 NFD cumulative release (%) profiles the three γ-CDPVAHMA formulations offered a burst release that lasted approximately 2 h, at which 59.1%, 51.9% and 46.8% of the NFD had been released from γ-CDPVAHMA1, γ-CDPVAHMA2 and γ-CDPVAHMA3 hydrogels, respectively. In accordance with previous reports, in the first part of release, the “burst effect” of NFD release was produced [17,51]. Possibly, this quick release is due to the remaining free NFD molecules around the hydrogel surface. After this fast release phase, a slower and steadier stage of NFD release into the medium called zero-order for all three cases of γ-CDPVAHMAs is produced. On the other hand, when the γ-CD content in the formulation increased, the release speed reduced, as depicted in Figure 8. This could be attributed to different factors that can involve the creation of a complex between γ-CDPVAHMAs and the drug; for instance, van der Waals interactions among the hydrophobic moiety of the NFD molecules and hydrophobic γ-CD cavity in γ-CDPVAHMAs. In addition, hydrogen bonding between the polar functional groups contained in both NFD and γ-CDPVAHMAs were identified. Additionally, the polar moieties of NFD and high-energy water molecules could form hydrogen bonds between during complex formation [24,52]. The NFD cumulative release (%) profile at pH 7.0 is depicted in Figure 8b for all three formulations. The γ-CDPVAHMAs offered a burst release into the medium up to a release time of 4 h, at which 83.90%, 74.4% and 67.9% of the NFD had been released from γ-CDPVAHMA1, γ-CDPvHMA2 and γ-CDPVAHMA3 hydrogels, respectively. If we compare the release at pH 7.4 with pH 3.0, the burst effect was greater and the zero-order stage was reached after 4 h. These results are in concordance with the swelling degree, given that at pH 7.4 the pendant groups from the hydrogel are ionized, developing fixed charges on the hydrogel.

Consequently, the hydrogel becomes more hydrophilic and it is expanded. Thus, these parameters at pH 7.4 could allow molecules flow faster from the hydrogel matrix to the outside. Hence, with the exclusive and tunable features of γ-CDPVAHMAs in relation with γ-CD content and the type of the designed hydrogel, the unique drug release profiles obtained for the present formulations denote their value as a possible controlled drug delivery system. These data are interesting in the context that we have obtained a formulation with singular characteristics to be used as a hypothetic oral dosage system of NFD.

### 3.7. Statistical Analysis for Release of NFD by γ-CDPVAHMAs

Table 5 shows the results of NFD release experiments.

**Figure 9:**
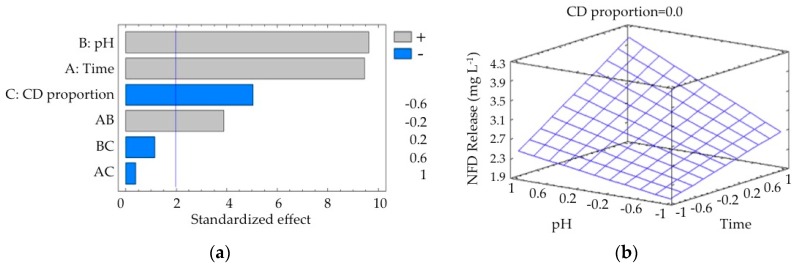
(**a**) Standardized Pareto chart for NFD release due to hydrogel treatment (A, time; B, pH; C, γ-CD proportion; AB, BC, and AC, interactions; and the line represents the critical *t*-value, 95% confidence); and (**b**) estimated response surface.

The release behavior of NFD from the formulations in PBS solution is presented in Figure 9. The Pareto chart (Figure 9a) shows that pH, time, hydrogel composition, and interaction between pH and time are statistically significant. Time and pH, and the interaction between pH and time exert a positive influence on the release of NFD, while the proportion of γ-CD in the hydrogel exerts a negative influence. As observed in Figure 9b, the estimated response surface shows that the release of NFD increased when the pH and time rose, reaching a maximum value at the end of each interval.

The regression equation of the model is described as Equation (5):

(5) NFD Release=2.80+0.578×A+0.473×B−0.304×C+0.238×A×B (R2=51.26)

To ensure the release of NFD for a longer time at physiological pH, the lowest proportion of γ-CD in the formulations should be used. Hypothetically, at pH 3.0, the NFD release from the formulation is lower than at pH 7.4. Therefore, the hydrogel would pass through the stomach releasing less NFD (hydrogel contracted), and, upon reaching the intestine, as the pH increases close to 8.0, a greater release of the drug (hydrogel expanded) would occur.

### 3.8. Thermogravimetric Analysis Results

**Figure 10:**
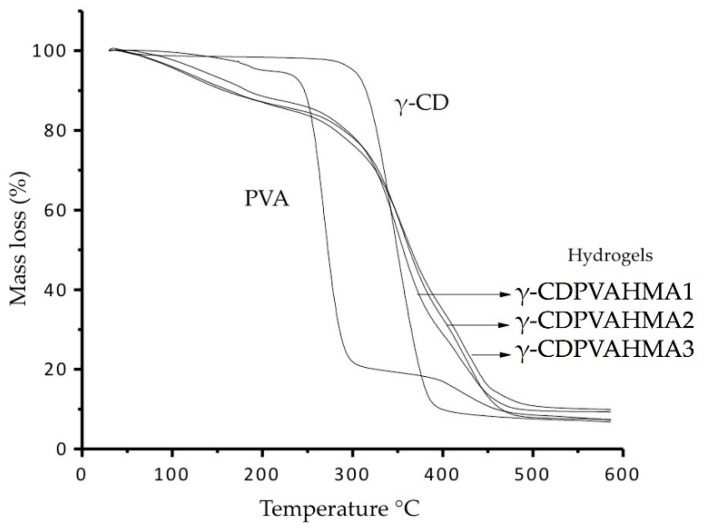
Thermograms of PVA, γ-CD, γ-CDPVAHMA1, γ-CDPVAHMA2, and γ-CDPVAHMA3.

The TGA analyses of the starting materials (pure PVA and pure γ-CD) and of the γ-CDPVAHMA formulations were performed. The results are shown in Figure 10 where the γ-CD mass loss with a maximum decomposition at 357 °C was observed. Furthermore, The PVA decomposition at a maximum temperature of 280 °C was detected. A second minority decomposition at a temperature near 350 °C corresponding to PVA was observed.

With respect to the thermogram of γ-CDPVAHMAs, the thermal stability of the formation of these formulations is greater than the thermic stability of the starting material compounds (PVA and γ-CD). In all thermograms, two temperatures or transition regions were observed. The first region (between 50 and 180 °C) is due to the loss of moisture and physically weak and chemically strong bound water. The second region with temperatures above 200 °C is attributed to the secondary degradation of free PVA and y-CD, corresponding to the inclusion complex and the synthetized formulations. These results conclude that there is an upper thermal stability for all the formulations due to the presence of chemical bonds among the PVA, MA, and γ-CD [53].

### 3.9. DTG Curves and Deconvolution Analysis

**Figure 11:**
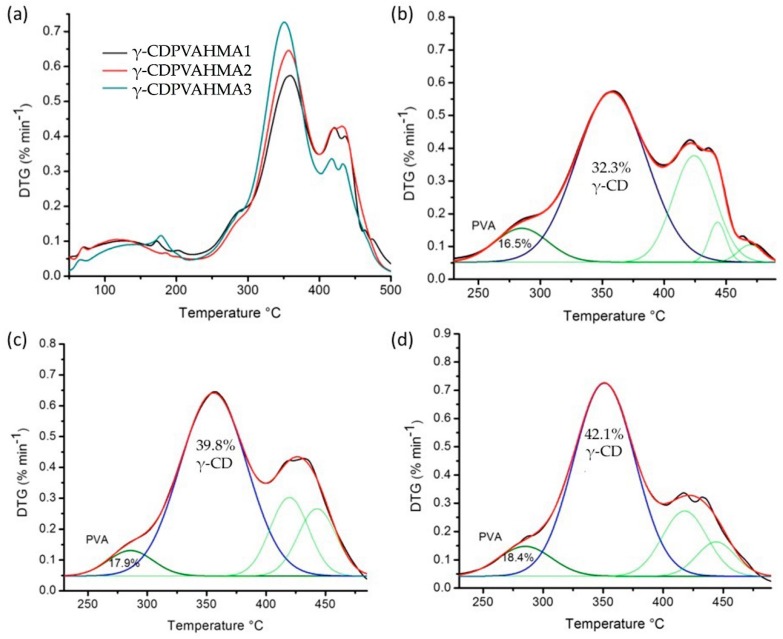
(**a**) DTG curves of γ-CDPVAHMAs and DTG and deconvolution curves of γ-CDPVAHMAs between 230 °C and 500 °C; (**b**) γ-CDPVAHMA1; (**c**) γ-CDPVAHMA2; and (**d**) γ-CDPVAHMA3.

In the present study, the DTG curves were investigated, as shown in Figure 11. Furthermore, a comparison of principal components of formulations through curves deconvolution was carried out. The relative area of the second region between 230 °C and ~500 °C (see Figure 11) corresponds to the components with major thermic stability, where five areas with Gaussian peaks were found for γ-CDPVAHMA1 and four for γ-CDPVAHMA2 and γ-CDPVAHMA3. The first and second peaks are attributed to free PVA (maximum temperature of degradation 280 °C) and free γ-CD (maximum temperature of degradation 350 °C), respectively. On the other hand, the results show that, for each formulation where different concentration (2.43 wt%, 3.61 wt% and 4.76 wt%) were added, there was also an increase in the percentage of concentration of γ-CD within of hydrogels. Besides, the relation between free PVA and free γ-CD decreased (0.438 of γ-CDPVAHMA1, 0.449 of γ-CDPVAHMA2, and 0.523 of γ-CDPVAHMA3), as depicted in Figure 11. This may be because of the cavities saturation that allows the occlusion within the hydrogel.

### 3.10. FT-IR Results

**Figure 12:**
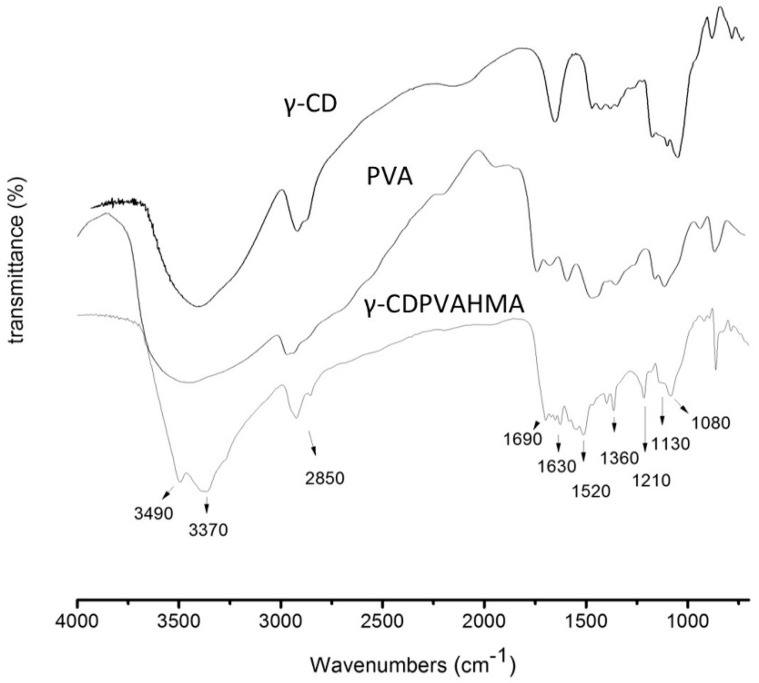
FT-IR spectra of typical γ-CDPVAHMA and starting material γ-CD and PVA.

As is reported in Figure 12, a signal for γ-CD at 3370 cm^−1^ was observed due to symmetric stretching of O–H at 2920 cm^−1^. Other signals that included symmetric stretching of –CH_2_ at 1210 cm^−1^ (antisymmetric stretching of C–C) and signal bending vibration of O–H at frequencies near at 1000 cm^−1^ were evidenced. Moreover, the typical PVA spectrum signal was detected, for instance, a signal C–H broad alkyl stretching band to 2850 cm^−1^, a characteristic hydrogen bonded band of O–H between 3200 and 3500 cm^−1^ and absorption peaks at 1080 and 1130 cm^−1^ were observed. The FTIR spectra of γ-CD and PVA are described in Figure 12. These last vibrational bands were attributed to stretching band C–O and C–C of the PVA, respectively [54,55]. As shown in Figure 12, a signal from γ-CD at 3370 cm^−1^ that is assigned to symmetric stretching of O–H at 2920 cm^−1^ was observed. Other signals were detected including symmetric stretching of –CH_2_ at 1210 cm^−1^ (antisymmetric stretching of C–C) and signal bending vibration of O–H at frequencies near 1000 cm^−1^. Moreover, the typical signals of PVA spectrum were observed, for instance, a signal C–H broad alkyl stretching band at 2850 cm^−1^ and a characteristic hydrogen bonded band from O–H between 3200 and 3500 cm^−1^. Moreover, it was possible to observe an absorption peak at 1080 and 1130 cm^−1^ that was attributed to a stretching vibrational band of C–O in PVA (Ricciardi, 2004; Mansur, 2004). The γ-CDPVAHMA formation signals were demonstrated for the intensive OH-group stretching that was revealed between 3200 and 3500 cm^−1^ (O–H asymmetrical and symmetrical stretching vibrations) and the deformation vibrations at 1630 cm^−1^ (H–O–H). Furthermore, the above mentioned may be due to the presence of water molecules in the network of the formulation (hydration of hydrogel) [56]. Finally, a signal at 1690 cm^−1^ was detected that corresponds to the covalent ester bonds formed between PVA chains and MA (crosslinking molecule), as well as between MA (partially bound at one end with PVA) and the -OH groups of γ-CD.

### 3.11. Evaluation of γ-CDPVAHMAs Cytotoxicity

**Figure 13:**
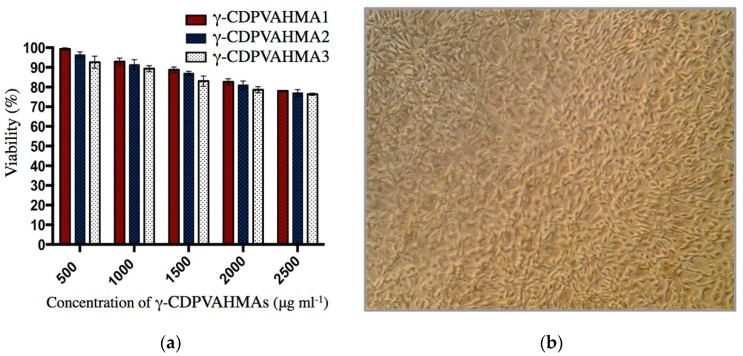
(**a**) Ratio (%) of cell viability, acquired from the MTT assay of the L929 fibroblast cells with respect to a negative control (without formulations); and (**b**) photograph of fibroblast cells cocultured with 2500 μg mL^−1^ of γ-CDPVAHMA3 (magnification 100×).

The MTT assay protocol was carried out to measure cell proliferation or cell cytotoxicity in fibroblast. The cytotoxic effect of the γ-CDPVAHMA1, γ-CDPVAHMA2, and γ-CDPVAHMA3 was evaluated by cell viability assay using L929 fibroblasts cells. Figure 13a shows fibroblast cell viability cocultured with different concentrations of γ-CDPVAHMAs (500–2500 μg mL^−1^) for 24 h. As displayed in Figure 13a, with an abrupt increase of the hydrogel amount, fibroblast cell viability reduced faintly, the viability ranging between 100% and 75%. Thus, the γ-CDPVAHMAs were able to maintain cell viability over 75%, although they were exposed to high concentrations of formulations. Figure 13b illustrates a microphotograph of fibroblasts cocultured with 2500 μg mL^−1^ of γ-CDPVAHMA3 formulation, where a high cell proliferation was detected. The cell viability analysis deduced that the formulations synthesized were biocompatible with low cell cytotoxicity. Consequently, these platforms based on hydrogels could be considered as a safe drug delivery system in relation to biocompatibility.

## 4. Conclusions

Through computer methodologies, it was possible to model and simulate different PVA hydrogels crosslinked with dicarboxylic acids that also contain γ-CD. These systems were designed as platforms for the sustained and controlled release of NFD at acidic and basic pH. According to the interaction energies calculated, maleic acid was selected as the candidate to carry out the crosslinking of the PVA hydrogel.

MD simulation studies allowed the characterization of the intermolecular interactions between NFD and γ-CDPVAHMA hydrogel formulations at acid and neutral-basic pH. The MD simulation showed that γ-CDPVAHMA at acid pH has a compact structure due to the not linked protonated carboxyl groups of MA being able to generate hydrogen bonds with the other MA connected to the PVA chains, resulting in stabilization and structural compacting of the hydrogel due to the attraction forces between PVA chains. When the hydrogel is subjected to a more basic environment, it undergoes a conformational change caused mainly by the deprotonation of the carboxyl groups, generating a greater separation between the PVA chains and, at the same time, its affinity for water molecules increases. The polymer network absorbs water through hydrogen bonds formed between water molecules and the deprotonated carboxyl groups. The conformational change and variation of hydrophilicity that the hydrogel undergoes allows the NFD molecules to be released selectively at a neutral-basic pH.

ESR and the thermomechanical properties from γ-CDPVAHMAs can be regulated by changing the γ-CD content. Higher γ-CD content in the hydrogels was associated with a higher concentration of loaded NFD. When the γ-CD content in the hydrogel increased, the release speed decreased. This could be due to multiple types of intermolecular interactions involved in the complex formation of γ-CDPVAHMAs with NFD. It is important to note that increasing the γ-CD content increased the hydrophilicity of the hydrogels. Therefore, the loading process and release of NFD could to be controlled by several factors: type of crosslinker, the crosslinking degree, number and size of pores, γ-CD content, and types of NFD-hydrogel intermolecular interactions.

The hydrogels showed good biocompatibility with L929 mouse connective tissue fibroblasts. In this study, the data concluded that stimuli-responsive hydrogels (swelling index) could change their volume significantly in response to small changes of certain environmental parameters such as time and pH. At physiological pH, it was observed that γ-CD content influenced the swelling index. In addition, the statistical analysis showed that γ-CD content influenced the percentage of NFD retention. Since these formulations possess excellent mechanical properties, low cytotoxicity and they can be tunable according to the drug release requirements, they may be utilized as an effective platform for NFD release to treat cardiological diseases.

## Figures and Tables

**Table 1:** Chemical structure and properties of Nifedipine (NFD) [4].

Chemical Characteristics and Properties	Detail	
Chemical Structure	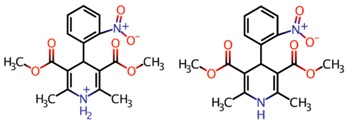 At acid pH At neutral and basic pH	
Molecular formula	C_17_H_19_N_2_O_6_	C_17_H_18_N_2_O_6_
Appearance	Yellow crystals	
Solubility in water (20–25 °C)	Insoluble	
Mol. Wt.	346.339 g mol^−1^	
Melting point	172–174 °C	
Wavelength (λ, nm)	240 nm	

**Table 2:** Components of the formulations of γ-CDPVAHMAs.

Hydrogel Formulation	γ-CD Proportion (%)	Copolymer Concentration % PVA/MA *w*/*w*	Hydrogel at 25 °C
γ-CDPVAHMA1	2.43	20%	Yes
γ-CDPVAHMA2	3.61	20%	Yes
γ-CDPVAHMA3	4.76	20%	Yes

**Table 3:** Average Δ*E* values calculated using SQM methods between PVAchain-DCAs-γCD block and NFD.

Id.	Hydrogel Block	Block/NFD Average Δ*E* at pH 3.0 kcal mol^−1^	Block/NFD Average Δ*E* at pH 7.4 kcal mol^−1^	Difference of Average Δ*E* pH 3.0–pH 7.4 kcal mol^−1^
1	PVAchain-Oxalic Acid-γCD	−2.99 ± 0.02	−2.38 ± 0.03	−0.61
2	PVAchain-Malonic Acid-γCD	−3.15 ± 0.04	−2.45 ± 0.02	−0.70
3	PVAchain-Succinic Acid-γCD	−3.30 ± 0.02	−2.41 ± 0.04	−0.89
4	PVAchain-Malic Acid-γCD	−3.76 ± 0.04	−2.39 ± 0.04	−1.37
5	PVAchain-Fumaric Acid-γCD	−3.21 ± 0.02	−2.34 ± 0.03	−0.87
6	PVAchain-Maleic Acid-γCD	−3.98 ± 0.04	−2.01 ± 0.02	−1.97
7	PVAchain-Citraconic Acid-γCD	−3.25 ± 0.03	−2.38 ± 0.04	−0.87
8	PVAchain-Itaconic Acid-γCD	−3.61 ± 0.04	−2.45 ± 0.02	−1.16
9	PVAchain-Tartaric Acid-γCD	−2.96 ± 0.03	−2.13 ± 0.05	−0.83
10	PVAchain-Glutaric Acid-γCD	−2.99 ± 0.02	−2.54 ± 0.03	−0.45
11	PVAchain-Adipic Acid-γCD	−3.10 ± 0.02	−2.63 ± 0.03	−0.47
12	PVAchain-Pimelic Acid-γCD	−3.25 ± 0.05	−2.87 ± 0.02	−0.38
13	PVAchain-Suberic Acid-γCD	−3.33 ± 0.03	−2.89 ± 0.04	−0.44
14	PVAchain-Azelaic Acid-γCD	−3.42 ± 0.03	−2.95 ± 0.05	−0.47
15	PVAchain-Phthalic Acid-γCD	−3.67 ± 0.02	−2.98 ± 0.02	−0.69
16	PVAchain-Isophthalic Acid-γCD	−3.75 ± 0.04	−2.75 ± 0.03	−1.00
17	PVAchain-Terephthalic Acid-γCD	−3.79 ± 0.05	−2.89 ± 0.04	−0.90
18	PVAchain-2,5-pyridine Acid-γCD	−3.68 ± 0.06	−2.45 ± 0.05	−1.23
19	PVAchain-Aspartic Acid-γCD	−3.98 ± 0.02	−3.69 ± 0.03	−0.29
20	PVAchain-Glutamic Acid-γCD	−3.87 ± 0.03	−3.65 ± 0.02	−0.22

**Table 4:** Amount of NFD loaded into the γ-CDPVAHMA formulations. Results indicate the average (*n* = 3) ± standard deviation values. The same letters beside the standard deviation denotes the absence of statistical differences using Tukey HSD, at 95% confidence level).

Composite	Amount of loaded NFD (mg g Dried Hydrogel^−1^) Concentration of Aqueous Soaking Solution 0.08 mg mL^−1^
γ-CDPVAHMA1	4.02 ± 0.32a
γ-CDPVAHMA2	4.49 ± 0.49a
γ-CDPVAHMA3	5.01 ± 0.5a

**Table 5:** Experimental design matrix. The values between parenthesis are the codified values.

Time of Release (h)	pH	γ-CD proportion (%)	NFD Release (mg L^−1^ ± SD) (*n* = 3)
0 (−1)	3 (−1)	2.43 (−1)	ND *
0.5 (−0.979)	3 (−1)	2.43 (−1)	1.94 ± 0.09
1 (−0.958)	3 (−1)	2.43 (−1)	2.10 ± 0.11
1.5 (−0.938)	3 (−1)	2.43 (−1)	2.22 ± 0.10
2 (−0.917)	3 (−1)	2.43 (−1)	2.34 ± 0.11
3 (−0.875)	3 (−1)	2.43 (−1)	2.44 ± 0.11
4 (−0.833)	3 (−1)	2.43 (−1)	2.49 ± 0.07
5 (−0.792)	3 (−1)	2.43 (−1)	2.49 ± 0.09
7 (−0.708)	3 (−1)	2.43 (−1)	2.57 ± 0.15
9 (−0.625)	3 (−1)	2.43 (−1)	2.57 ± 0.06
24 (0)	3 (−1)	2.43 (−1)	2.61 ± 0.10
30 (0.25)	3 (−1)	2.43 (−1)	2.65 ± 0.09
48 (1)	3 (−1)	2.43 (−1)	2.64 ± 0.13
0 (−1)	3 (−1)	3.61 (0.0129)	ND *
0.5 (−0.979)	3 (−1)	3.61 (0.0129)	1.59 ± 0.08
1 (−0.958)	3 (−1)	3.61 (0.0129)	1.86 ± 0.06
1.5 (−0.938)	3 (−1)	3.61 (0.0129)	1.94 ± 0.05
2 (−0.917)	3 (−1)	3.61 (0.0129)	2.06 ± 0.08
3 (−0.875)	3 (−1)	3.61 (0.0129)	2.22 ± 0.11
4 (−0.833)	3 (−1)	3.61 (0.0129)	2.30 ± 0.21
5 (−0.792)	3 (−1)	3.61 (0.0129)	2.34 ± 0.11
7 (−0.708)	3 (−1)	3.61 (0.0129)	2.44 ± 0.09
9 (−0.625)	3 (−1)	3.61 (0.0129)	2.47 ± 0.11
24 (0)	3 (−1)	3.61 (0.0129)	2.53 ± 0.11
30 (0.25)	3 (−1)	3.61 (0.0129)	2.51 ± 0.12
48 (1)	3 (−1)	3.61 (0.0129)	2.51 ± 0.06
0 (−1)	3 (−1)	4.76 (1)	ND *
0.5 (−0.979)	3 (−1)	4.76 (1)	1.25 ± 0.13
1 (−0.958)	3 (−1)	4.76 (1)	1.61 ± 0.09
1.5 (−0.938)	3 (−1)	4.76 (1)	1.78 ± 0.11
2 (−0.917)	3 (−1)	4.76 (1)	1.85 ± 0.12
3 (−0.875)	3 (−1)	4.76 (1)	1.96 ± 0.08
4 (−0.833)	3 (−1)	4.76 (1)	1.99 ± 0.11
5 (−0.792)	3 (−1)	4.76 (1)	2.03 ± 0.15
7 (−0.708)	3 (−1)	4.76 (1)	2.11 ± 0.11
9 (−0.625)	3 (−1)	4.76 (1)	2.14 ± 0.10
24 (0)	3 (−1)	4.76 (1)	2.16 ± 0.18
30 (0.25)	3 (−1)	4.76 (1)	2.18 ± 0.11
48 (1)	3 (−1)	4.76 (1)	2.16 ± 0.07
0 (−1)	7.4 (1)	2.43 (−1)	ND *
0.5 (−0.979)	7.4 (1)	2.43 (−1)	1.94 ± 0.07
1 (−0.958)	7.4 (1)	2.43 (−1)	2.50 ± 0.17
1.5 (−0.938)	7.4 (1)	2.43 (−1)	2.62 ± 0.11
2 (−0.917)	7.4 (1)	2.43 (−1)	2.80 ± 0.13
3 (−0.875)	7.4 (1)	2.43 (−1)	3.12 ± 0.11
4 (−0.833)	7.4 (1)	2.43 (−1)	3.32 ± 0.10
5 (−0.792)	7.4 (1)	2.43 (−1)	3.47 ± 0.10
7 (−0.708)	7.4 (1)	2.43 (−1)	3.67 ± 0.10
9 (−0.625)	7.4 (1)	2.43 (−1)	3.80 ± 0.11
24 (0)	7.4 (1)	2.43 (−1)	3.95 ± 0.08
30 (0.25)	7.4 (1)	2.43 (−1)	3.93 ± 0.13
48 (1)	7.4 (1)	2.43 (−1)	3.96 ± 0.13
0 (−1)	7.4 (1)	3.61 (0.0129)	ND *
0.5 (−0.979)	7.4 (1)	3.61 (0.0129)	1.48 ± 0.12
1 (−0.958)	7.4 (1)	3.61 (0.0129)	1.95 ± 0.07
1.5 (−0.938)	7.4 (1)	3.61 (0.0129)	2.32 ± 0.02
2 (−0.917)	7.4 (1)	3.61 (0.0129)	2.55 ± 0.12
3 (−0.875)	7.4 (1)	3.61 (0.0129)	2.79 ± 0.09
4 (−0.833)	7.4 (1)	3.61 (0.0129)	2.94 ± 0.09
5 (−0.792)	7.4 (1)	3.61 (0.0129)	3.10 ± 0.09
7 (−0.708)	7.4 (1)	3.61 (0.0129)	3.28 ± 0.11
9 (−0.625)	7.4 (1)	3.61 (0.0129)	3.38 ± 0.16
24 (0)	7.4 (1)	3.61 (0.0129)	3.58 ± 0.14
30 (0.25)	7.4 (1)	3.61 (0.0129)	3.63 ± 0.09
48 (1)	7.4 (1)	3.61 (0.0129)	3.65 ± 0.09
0 (−1)	7.4 (1)	4.76 (1)	ND *
0.5 (−0.979)	7.4 (1)	4.76 (1)	1.18 ± 0.06
1 (−0.958)	7.4 (1)	4.76 (1)	1.69 ± 0.14
1.5 (−0.938)	7.4 (1)	4.76 (1)	1.97 ± 0.05
2 (−0.917)	7.4 (1)	4.76 (1)	2.27 ± 0.06
3 (−0.875)	7.4 (1)	4.76 (1)	2.51 ± 0.10
4 (−0.833)	7.4 (1)	4.76 (1)	2.69 ± 0.12
5 (−0.792)	7.4 (1)	4.76 (1)	2.88 ± 0.07
7 (−0.708)	7.4 (1)	4.76 (1)	3.03 ± 0.16
9 (−0.625)	7.4 (1)	4.76 (1)	3.08 ± 0.07
24 (0)	7.4 (1)	4.76 (1)	3.13 ± 0.09
30 (0.25)	7.4 (1)	4.76 (1)	3.14 ± 0.11
48 (1)	7.4 (1)	4.76 (1)	3.14 ± 0.12

* ND, Not Detected.

## Supplementary Materials

The following are available online at http://www.mdpi.com/2073-4360/10/7/806/s1, Figure S1: SASA and NFD retention plots, Script S1: TCL script to calculate the Radius of Gyration (RGYR), Script S2: TCL script to calculate the Solvent Accessible Surface Area (SASA), Script S3: TCL script to calculate the number of NFD molecules located within 5.5 Å of the hydrogel.
