# Supplementary material for: Rational Development of a Novel Hydrogel as a pH-Sensitive Controlled Release System for Nifedipine
Source: Polymers (Basel). 2018 Jul 23;10(7):806. doi: 10.3390/polym10070806 (PMC6403543; doi:10.3390/polym10070806)
Supplement: Supplementary file 1 [file polymers-10-00806-s001.pdf]

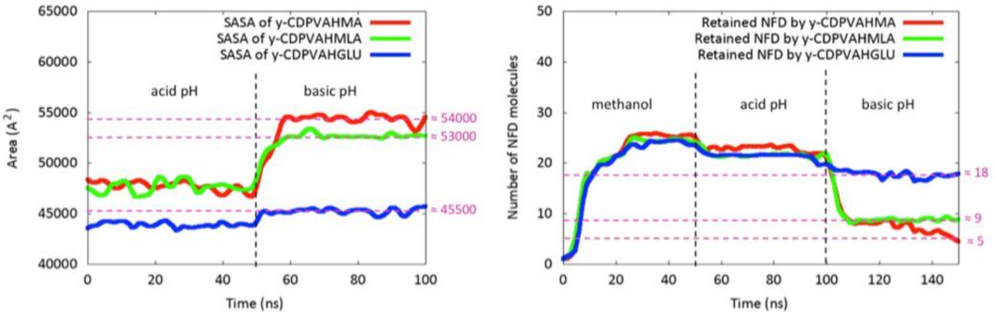

(**a**) (**b**)

Figure S1. (a) Solvent Accessible Surface Area (SASA) plots of the hydrogels crosslinked with maleic acid (γ-CDPVAHMA), malic acid (γ-CDPVAHMLA) and glutamic (γ-CDPVAHGLU), at acid (0-50 ns) and neutral-basic pH (51-100 ns), respectively. (**b**) Number of NFD molecules retained by γ-CDPVAHMA, γ-CDPVAHMLA and γ-CDPVAHGLU during the three simulations: in methanol (0-50 ns), at acid pH (51-100 ns) and at neutral-basic pH (101-150 ns).
